# The Potential Role of Oxidative Stress in Modulating Airway Defensive Reflexes

**DOI:** 10.3390/antiox14050568

**Published:** 2025-05-09

**Authors:** Yuki Sato, Yoichiro Sugiyama, Tomoya Ishida, Haruhiko Inufusa, Fukka You, Davis Joseph, Shigeru Hirano

**Affiliations:** 1 Department of Otolaryngology-Head and Neck Surgery, Faculty of Medicine, Saga University, 5-1-1, Nabeshima, Saga 849-8501, Japan; ss4436@cc.saga-u.ac.jp (Y.S.); sr1360@cc.saga-u.ac.jp (T.I.); 2 Division of Anti-Oxidant Research, Life Science Research Center, Gifu University, Yanagito 1-1, Gifu 501-1194, Japan; hinufusa@gmail.com (H.I.); y@antioxidantres.jp (F.Y.); 3 Anti-Oxidant Research Laboratory, Louis Pasteur Center for Medical Research, Tanaka Monzen-cho 103-5, Sakyo-ku, Kyoto 606-8225, Japan; 4 Faculty of Medicine, McGill University, Montreal, QC H3A 0G4, Canada; 5 Flogen Technologies Inc., Mount Royal, QC H3P 2T1, Canada; 6 Department of Otolaryngology-Head and Neck Surgery, Kyoto Prefectural University of Medicine, 465 Kajii-cho, Kamigyo-ku, Kyoto 602-8566, Japan; hirano@koto.kpu-m.ac.jp

**Keywords:** oxidative stress, swallowing, coughing, airway defensive reflex

## Abstract

Airway defensive reflexes, such as pharyngeal swallowing, coughing, and sneezing, play a pivotal role in maintaining airway homeostasis. These reflexes are controlled by complex mechanisms primarily governed by specific neuronal circuitry in the brainstem, referred to as central pattern generators. These behaviors also require optimal conditions for the peripheral organs within the airway and alimentary tracts, including the nose, pharynx, larynx, and trachea, which are vital for ensuring appropriate responsiveness and motor outputs. Oxidative stress is linked to the development and progress of impaired functions of those behaviors. Dysphagia caused by central or peripheral impairments, such as neurodegeneration of related neuronal networks and laryngeal desensitization, is likely associated with an increased level of oxidative stress. Chronic inflammation and allergic airway sensitization in the lower airways, including asthma, elevate oxidative stress levels and diminish the activity of antioxidant defense enzymes, which exacerbate the severity of respiratory conditions. Antioxidant supplements offer promising therapeutic benefits by facilitating the recovery of distorted airway defensive reflexes, although limited information has been provided concerning therapeutic strategies. Further studies are necessary to enhance our understanding of the pathophysiology of dysphagia and airway diseases related to oxidative stress, as well as to develop new treatment strategies for these disorders.

## 1. Introduction

**Figure 1:**
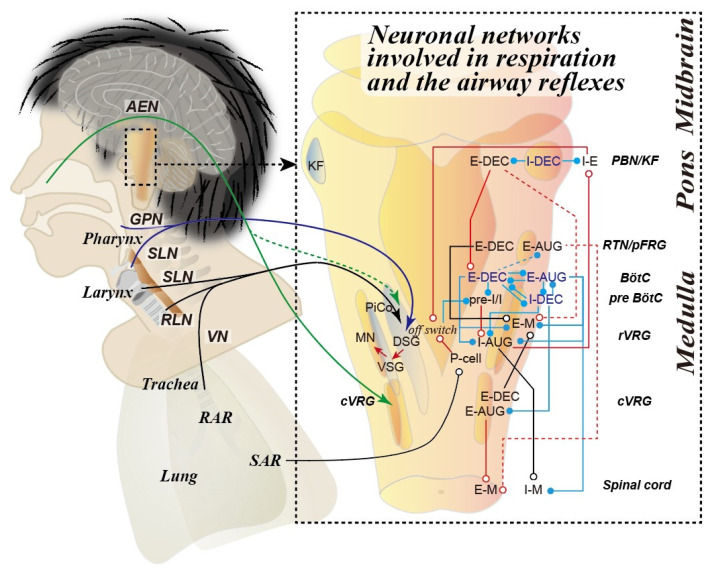
The neuronal networks involved in respiration and the airway defensive reflexes including swallowing, coughing, and sneezing [2,15,16,17,18,19,20,21,22]. Inhibitory and excitatory interconnections among respiratory-related neurons in the pontomedullary respiratory columns are depicted. Respiratory rhythmogenesis is controlled by various kinds of respiratory-related neurons. Sensory signals eliciting swallowing (blue arrow), coughing (black arrow), and sneezing (green arrow) are illustrated in the left illustration. Inhibitory and excitatory inputs are depicted by closed and open circles, respectively. Augmenting expiratory neuron, E-AUG; augmenting inspiratory neuron, I-AUG; decrementing expiratory neuron, E-DEC; decrementing inspiratory neuron, I-DEC; expiratory motoneuron, E-M; inspiratory motoneuron, I-M; pre-inspiratory/inspiratory neuron, pre-I/I; pump neuron, P-cell; inspiratory–expiratory neuron, I-E; dorsal swallowing group, DSG; ventral swallowing group, VSG; cranial motoneuron, MN; post-inspiratory complex, PiCo; caudal ventral respiratory group, cVRG; rostral ventral respiratory group, rVRG; pre-Bötzinger complex, pre-BötC; Bötzinger complex, BötC; retrotrapezoid/parafacial respiratory group, RTN/pFRG; parabrachial/Kölliker–Fuse nuclei, PBN/KF, anterior ethmoidal nerve, AEN; glossopharyngeal nerve, GPN; superior laryngeal nerve, SLN; recurrent laryngeal nerve, RLN; vagus nerve, VN; rapidly adapting receptor, RAR; slowly adapting receptor, SAR.

Airway defensive reflexes, including pharyngeal swallowing, coughing, and sneezing, are crucial for maintaining airway clearance and patency during respiration, eating, and speaking. These behaviors consist of well-organized and optimized motor patterns of the oropharyngeal, laryngeal, and other respiratory muscles. Eating involves several stages: oral preparation, transferring the bolus to the pharynx, and subsequent oropharyngeal swallowing. The pharyngeal stage of swallowing consists of a coordinated sequence of muscle relaxations and contractions, which produce laryngeal elevation, closure of the nasopharynx, constriction of the pharynx, glottal closure, relaxation of the cricopharyngeal muscle, and esophageal peristalsis [1,2]. All of these activities are seamlessly coordinated and controlled by neuronal networks within the central nervous system (CNS) (Figure 1) [3,4,5,6]. In particular, pharyngeal swallowing is essential for effective food transfer to the esophagus while also preventing aspiration. This process involves complex and coordinated movements of the pharynx and larynx [7,8,9]. If penetration or aspiration occurs, foreign substances in the airway should be promptly expelled from the larynx and lower airway.

Coughing is a critical defensive reflex that removes foreign materials and secretions in the airway [10]. Excessive sensitization of the respiratory tract due to allergies and chronic inflammation can lead to severe and repeated coughing, which may deteriorate respiratory conditions [11,12,13].

Sneezing is an upper airway reflex that expels foreign bodies through the nose and throat [10]. Nasal immune hyper-reactivity characterized by Th2-dominant allergic inflammation in response to various allergens, such as house dust and pollens, is enhanced by several cytokines, including interleukin (IL)-4, IL-5, and IL-13 released by specific immune cells [14]. These immune responses increase the sensitivity of the nasal sensory nerves, leading to excessive sneezing.

Oxidative stress and oxidative/antioxidative imbalance negatively impact those pathological conditions. In this review, we focus on the pathophysiological role of oxidative stress in the development and progression of airway defensive reflex impairment and highlight the potential significance of antioxidants for ameliorating those functions.

## 2. Neuronal Mechanisms Underlying Respiration and Airway Protective Reflexes

The coordination of breathing and digestion plays a crucial role in facilitating effective gas exchange in the lungs and ensuring proper airway clearance throughout both the upper and lower airways [23,24,25,26]. These airway systems are manipulated by sophisticated and complex movements of the airway and alimentary tract consisting of the intrinsic and extrinsic muscles of the tongue, which facilitate speech and food manipulation; the internal and external laryngeal muscles, which are essential for voice production and airway defense; the pharyngeal constrictor muscles, which help bolus transfer during swallowing; the esophageal peristaltic muscles, which produce peristaltic movements to facilitate food transfer to the stomach; and the diaphragm and abdominal muscles, which are vital for adequate lung inflation and deflation. These behaviors are intricately coordinated and regulated by specialized neuronal networks in the brain, referred to as central pattern generators (CPGs) (Figure 1) [27], including respiratory CPG [15,28,29,30], swallowing CPG [2,31,32,33], coughing neuronal circuitry [16,34,35], and sneezing neuronal circuitry [17,36,37]. These neuronal networks are mainly distributed within the pontomedullary regions of the brain, highlighting the remarkable complexity of mutual interaction within the circuits [35,38,39,40].

Respiration can be classified into two phases: the inspiratory and expiratory phases. The early part of the expiratory phase is called the post-inspiratory phase, during which the slight adduction of the vocal folds occurs to prevent lung collapse and ensure efficient gas exchange [28,30,41,42]. Hence, normal respiration can be reclassified into three phases: the inspiratory, post-inspiratory, and late expiratory phases [28,30,41,42]. These phases are orchestrated by the respiratory CPG, which is located in the neural structures extending from the medulla oblongata to the pons. The respiratory CPG is composed of two primary neuronal columns: the dorsal and ventral columns [28]. The dorsal respiratory group, located ventrolaterally to the nucleus tractus solitarius (NTS), consists mainly of inspiratory neurons, some of which may also contribute to Schluckatmung [43]. In the ventral column, the arrangement of respiratory neurons follows a rostrocaudal distribution with distinct characteristics across different subgroups. The caudal ventral respiratory group is tasked with regulating abdominal contractions involved in breathing, coughing, sneezing, and vocalization [16,44,45,46]. Conversely, the rostral ventral respiratory group is responsible for generating inspiratory-related activity [15,40]. The pre-Bötzinger complex and the parafacial respiratory group function as the generation of inspiratory and expiratory rhythms, respectively [18,47,48]. Furthermore, the Kölliker–Fuse nucleus is essential for producing post-inspiratory activity in combination with the post-inspiratory complex [18,30]. Neuronal networks and interconnections among respiratory-related neurons in the pontomedullary respiratory columns are illustrated in Figure 1. These neuronal components have an indispensable role in the initiation and regulation of respiration–swallowing coordination. Optimized interaction between breathing and swallowing is required for airway management.

Pharyngal swallowing is a highly complex physiological process involving stereotyped and replicative movements of various swallowing-related muscles, and it differs from simple reflex actions [1,2]. The pharyngeal phase of swallowing and subsequent esophageal peristalsis occur involuntarily and are not subject to conscious control [31,32,49]. Most of the muscles involved in this process are managed by a neural circuitry that governs predetermined movements, ensuring a consistent response regardless of the specific characteristics of the bolus. This neural mechanism should function rapidly and synchronously with the bolus from the oral cavity to the esophagus via the pharynx to prevent aspiration. This mechanism also facilitates the removal of foreign objects from the lower airway when penetration and aspiration occur. It is also crucial to understand the mechanisms involved in cough generation. The swallowing CPG is primarily composed of two distinct but partially overlapping groups of neurons: the dorsal swallowing group (DSG) and the ventral swallowing group (VSG) [2,50]. DSG neurons are located in and around the NTS, with a subset of these neurons receiving sensory inputs from peripheral afferent pathways connected to the larynx and pharynx [51]. This sensory input plays a critical role in initiating and modulating the motor movements involved in swallowing. The significant function of the DSG is to establish the motor patterns necessary for the pharyngeal stage of swallowing, as well as to produce the subsequent peristaltic movements of the esophagus [2,52,53]. In contrast, VSG neurons are located in the vicinity of the nucleus ambiguus and act as premotor neurons that drive swallowing motor activity [2]. Pharyngeal swallowing is primarily triggered by sensory stimulation in the pharynx and larynx from a food bolus. In contrast, stimulating the glossopharyngeal nerve and the internal branch of the superior laryngeal nerve inhibits respiratory activity [54,55]. Consequently, these sensory stimuli that promote swallowing also influence the respiratory CPG [26,35,56]. However, once the pharyngeal swallowing process is initiated, the respiratory CPG optimizes swallowing by modulating the activity of various respiratory CPG neurons in synchrony with pharyngeal swallowing, which can help respiration–swallowing coordination to maintain breathing and eating functions [49]. The phase transitions from normal respiratory rhythm to pharyngeal swallowing and from resetting to eupneic respiratory patterns following the swallowing process are significant mechanisms that can diminish the risk of aspiration [57]. The initiation of pharyngeal swallowing typically occurs during the expiratory phase, followed by a reset into subsequent breathing that continues with expiration. The respiratory and swallowing CPGs provide mutual interaction, ensuring the proper initiation timing of swallowing, which allows for a smooth phase transition that does not disrupt pulmonary gas exchange. Swallowing initiation and adaptation to bolus characteristics can be optimized by ascending and descending signals to the higher centers in the suprabulbar region, such as the basal ganglia, amygdala, hypothalamus, pontine, cerebellum, anterior cingulate cortex, and cortical mastication area [2,58,59,60,61,62,63].

Aspiration can be developed by various pathogeneses, such as delayed laryngeal closure in response to the bolus transfer into the pharynx, which may be caused by the desensitization of the pharynx and larynx or decreased facilitatory signals from the higher centers to the swallowing CPG. Other contributing factors include restricted laryngeal movement following radiation therapy for head and neck cancers, incomplete glottal closure due to vocal fold paralysis, poor pharyngeal clearance due to decreased pharyngeal contractions or velopharyngeal insufficiency, impaired relaxation of the cricopharyngeal muscle, and increased resistance at the esophageal entrance due to inadequate laryngeal elevation [64]. Guillain–Barré syndrome is associated with a significant reduction in the motor output of both the pharynx and larynx, which can lead to swallowing difficulties. In addition, unilateral vagus nerve paralysis, possibly caused by the herpes zoster virus, may be attributed to vocal cord paralysis and impairs pharyngeal clearance due to reduced laryngeal and pharyngeal muscle contractions [65]. Similarly, myogenic diseases such as dermatomyositis can increase aspiration risks by disrupting pharyngeal contraction and impairing the elevation of the larynx [66].

To prevent aspiration and aspiration pneumonia, maintaining a robust cough reflex is essential, as it effectively removes foreign objects from the lower airway, facilitating airway clearance. Similarly to the process of swallowing, the cough reflex involves complex, coordinated movements governed by specific neural circuits that are located from the medulla oblongata to the pons and moderately overlapping the respiratory center [10,34]. This physiological reflex is important for maintaining airway health, optimizing respiratory function, and preventing the risk of aspiration pneumonia.

The coughing motor pattern consists of the following sequential phases: inspiratory, compressive, expulsive, and narrowing phases [10,67]. Following inhalation, glottal adduction occurs alongside the contraction of the abdominal muscles during the compressive phase. This motion elevates subglottic pressure, creating a sufficient force for effective expiration. During the expulsive phase, powerful contractions of the abdominal muscles, in accordance with the dilation of the glottis, facilitate a strong airflow that expels foreign substances from the respiratory tract through the mouth. After the expulsive phase of coughing, the vocal folds then adduct (i.e., the narrowing phase). The intricate coordination of laryngeal and abdominal muscle activities that precede inspiration is intricately regulated by synaptic pathways to the motoneurons innervating the respiratory and oropharyngeal muscles, including the phrenic, abdominal, and laryngeal motoneurons. This mechanism is regulated by the coughing neuronal circuitry composed of a subset of respiratory neurons and various nuclei located in the brainstem, which coordinate the complex motor responses associated with coughing [16,34,45]. Coughing can be triggered by irritation of laryngeal and tracheo-pulmonary afferents through the superior laryngeal, recurrent laryngeal, and vagus nerves, activating peripheral receptors like rapidly adapting and transient receptor potential vanilloid 1 (TRPV1) receptors [19,20]. Many previous reports about neural mechanisms that generate and control coughing showed that coughing can be triggered by laryngeal, tracheal, and pulmonary afferent stimulation and is classified into laryngeal and tracheobronchial coughing [10,11,13,28,35,38,67,68]. Consequently, brainstem neuronal networks widely overlapping with the respiratory CPG primarily generate the cough reflex [16,34,35,38,69]. In addition, the cough reflex could be modulated by other brainstem areas, including the lateral tegmental field, nucleus tractus solitarius, parabrachial nucleus, and cerebellum [70,71,72].

Sneezing is an essential protective reflex for the respiratory system, allowing for removing mucus and other secretions from the nasal passages and throat. Studies have highlighted the neural circuitry involved in the sneezing reflex [17,37,73,74,75]. This process can be initiated explicitly by irritation of the nasal mucosa, which sends signals to the caudal respiratory group, the NTS, and other brainstem nuclei related to respiration via the anterior ethmoidal nerve, leading to the production and regulation of sneezing [17,68,74,76]. Sneezing involves the activation of specific motor patterns within the phrenic, abdominal, and laryngeal muscles, which differ from those during coughing [17,67]. However, the trajectories of membrane potentials of the cranial motor and premotor neurons exhibit similar characteristics during these two behaviors [67,68]. Fine-tuning the optimization of coughing and sneezing may be accomplished through the intrinsic reconfiguration of the respiratory CPG and by mediating the interactions between the neuronal pathways responsible for coughing and sneezing and the respiratory CPG [17,34,36,40].

The neuronal mechanisms underlying airway protective reflexes, including coughing and sneezing, remain unrevealed. Nevertheless, it is likely that the adaptive reconfiguration of the respiratory CPG regulates the coordinated and predetermined activities of respiratory and oropharyngeal muscles and is modulated by some other brainstem regions [16,67,69,75,77,78]. Meanwhile, the swallowing CPG consists of broadly distributed neurons in the medulla, primarily organized into dorsal and ventral swallowing groups. In parallel, numerous respiratory neurons—including those in the Bötzinger complex and the dorsal and ventral respiratory groups—exhibit coordinated activation or inhibition during the swallowing process. This synchronization facilitates specific swallowing movements and plays a crucial role in the coordination between breathing and swallowing. Additionally, certain respiratory neurons actively contribute to generating swallowing motor activity. As a result, distinguishing between the respiratory CPG and the swallowing CPG becomes challenging, highlighting the intricate interplay between these systems in controlling these vital functions.

## 3. Functional Characteristics of Upper Airway Muscles Involved in Airway Reflexes

Airway reflexes are vital physiological responses that result from the coordinated and specific movement of respiratory-related muscles in the upper airway and alimentary tracts. Most of these muscles are controlled by cranial motor nerves, including the trigeminal, facial, glossopharyngeal, vagus, and hypoglossal nerves. The modulation of the membrane potential in the motoneurons that innervate the upper airway muscles (e.g., the swallowing-related muscles) is crucial for optimizing airway patency and defensive airway reflexes [79]. Many animal and human studies have revealed that many upper airway muscles are activated in synchrony with inhalation [80,81,82,83]. In contrast, some muscles, such as the pharyngeal constrictor, laryngeal adductor, and hyoglossus muscles, show activity associated with exhalation [9,84,85]. The activities of these cranial motoneurons are influenced by phasic signals generated through excitatory and inhibitory inputs from the respiratory CPG. This mechanism likely helps to prevent the disuse atrophy of these muscles [86].

During swallowing, the motoneurons controlling the pharyngeal constrictor muscles discharge action potentials in a specific sequence following a brief period of inhibition [9]. The initiation and duration of motor unit activity in these muscles (such as the thyropharyngeal and cricopharyngeal muscles) are regulated by both inhibitory and excitatory postsynaptic membrane potentials of the pharyngeal motoneurons. These activities create peristaltic waves in the pharynx to convey the bolus to the esophagus. Additionally, there is a brief suppression of activity prior to glottal closure during swallowing, which is also controlled by the swallowing CPG [87]. This temporary relaxation of the vocal folds may help prevent aerophagia during pharyngeal swallowing.

As described above, coughing is divided into following phases: inspiratory, compressive, and expulsive (and subsequent narrowing) phases [10,67]. The compressive phase is crucial in increasing subglottic pressure, which helps expel foreign materials and secretions from the trachea. To generate effective force prior to the expulsive phase, a strong and forceful contraction of the bilateral vocal fold adductor muscles and a strong contraction of the abdominal muscles are necessary [35].

Patterned movements of the respiratory and upper airway muscles during sneezing are similar to those during coughing [67]. Sneezing also involves inspiratory, compressive, and expulsive phases that generate glottal adduction prior to inhalation. This is followed by forced expiration combined with oropharyngeal activity to effectively expel foreign materials through the nose and throat.

The organization of the oropharyngeal and respiratory muscles is fundamentally managed by respiratory neuronal networks involving multifunctional premotor neurons within the brainstem [28,68]. During normal breathing, known as eupnea, the regulation of these muscles is predominantly governed by the respiratory CPG [28]. When the pharyngeal and laryngeal afferent signals stimulated by a bolus are infused into the oropharyngeal cavity and are accumulated over the threshold for activating the swallowing CPG, the sequential motor patterns of pharyngeal swallowing are initiated [2]. This activation leads to the reconfiguration of the respiratory CPG, promoting the coordinated motor pattern for pharyngeal swallowing [40]. These neuronal interactions play a crucial role in preventing aspiration and optimizing the timing of swallowing initiation during the ongoing respiratory cycle, which is capable of executing respiratory protection and efficient bolus transfer during the swallowing process. Coughing and sneezing are also well-organized oropharyngeal and respiratory muscle activities that involve distinct motor patterns triggered by irritation in the airways [9,10,37,67,75]. These motor patterns are primarily regulated by the reconfigured respiratory CPG and are fine-tuned for each specific behavior. In addition, proprioceptive and vagal afferent signals provide phase transitions during these reflexive movements to facilitate adequate rhythm generation during each reflex [22,34]. As such, the oropharyngeal muscles, such as the laryngeal and pharyngeal constrictor muscles, exhibit distinct characteristics compared to somatic skeletal muscles, reflecting their specialized roles in respiratory function [86].

Precise control of the activity of the laryngeal motoneurons is required to generate sneezing, swallowing, and coughing, along with synchronized activities of phrenic, abdominal, and hypoglossal motoneurons [17,67]. These multifunctional activities of the cranial motoneurons are regulated by the respiratory, swallowing, coughing, and sneezing neuronal circuits via premotor neurons in the brainstem nuclei (Figure 1). Further studies are needed to explore the interconnections to the motoneurons generating these multifunctional motor patterns.

## 4. The Role of Oxidative Stress in the Development and Deterioration of Airway Reflexes

Oxidative stress is intricately linked to mitochondrial dysfunction, which results in reduced ATP production and an elevated presence of free radicals in the brain [88,89,90]. Reactive oxygen species (ROS) are derivatives of oxygen that include free radicals such as nitric oxide, superoxide anion, hydroxyl radical, hydrogen peroxide, and hypochlorous acid [91,92]. These substances may cause cellular damage by oxidizing biomolecules. Continuous exposure to oxidative stress can result in significant DNA damage due to mitochondrial dysfunction, particularly affecting the genes and proteins encoded within the mitochondria [89,93,94,95]. DNA damage and the alteration of other biomolecules linked to oxidative stress are associated with the pathological factors of neurodegenerative diseases, such as Parkinson’s disease [96,97]. Reoxygenation during reperfusion after ischemic brain damage exposes cellular DNA, lipids, and proteins to ROS oxidation reactions [98,99]. Furthermore, antioxidative defense mechanisms mitigate superoxide radicals associated with ischemic brain damage, using endogenous antioxidants to scavenge certain free radicals [99]. Mitochondrial dysfunction exacerbates ROS production, contributing to severe cellular damage with implications for various diseases and aging [88,89]. Additionally, ROS produced by immune cells involved in host defense, including polymorphonuclear neutrophils, can progress inflammatory diseases through the oxidation of cellular signaling proteins, leading to endothelial dysfunction and tissue injury [92].

Oxidative stress influences respiration and airway defensive reflexes. Dysphagia is a common disorder that occurs following a stroke [3,62]. After ischemic stroke in the medulla oblongata, including the swallowing center (i.e., Wallenberg syndrome), severe swallowing impairment can occur contingent upon the rostrocaudal spread of the lesion [100]. As described above, the DSG governs swallowing initiation and motor pattern generation. Meanwhile, the cranial motor neurons innervating the swallowing muscles provide the essential motor output necessary for driving swallowing movements. Primary lateral sclerosis is an example of a specific neurodegenerative disease [101]. Functional deficits in the regulatory mechanisms of swallowing can lead to delayed swallowing initiation, insufficient opening of the esophageal entrance, and bulbar paralysis, which includes unilateral vocal fold and pharyngeal paralysis [102].

In addition, ischemic damage to the CNS results in neuronal deficits, which could significantly compromise brain function. The reperfusion phase following ischemic events can lead to additional functional and histological damage in the surrounding tissues, thereby exacerbating CNS dysfunction and possibly leading to medullary stroke [103,104]. Oxidative stress that occurs post-reperfusion contributes to neurodegenerative processes [105]. Many previous reports have indicated that oxidative stress significantly contributes to the progression of neurodegenerative diseases, including Parkinson’s disease and amyotrophic lateral sclerosis (ALS) [106,107,108,109,110,111]. In addition, oxidative stress leads to the enhanced generation of oxidatively modified α-synuclein, increased aggregation of α-synuclein into oligomeric species, and pronounced degeneration within and outside the CNS, including dopaminergic neurons in the substantia nigra pars compacta and the dorsal motor nucleus of the vagus nerve [106,107,112,113,114].

Aging can negatively impact swallowing function and cause decreased activity of swallowing-related muscles and the swallowing reflex, partially due to reduced levels of antioxidants [115,116]. Glottal insufficiency due to age-related atrophy of the vocal folds may impact pressure generation in the pharynx during swallowing, potentially increasing the risk of aspiration [117,118].

**Figure 2:**
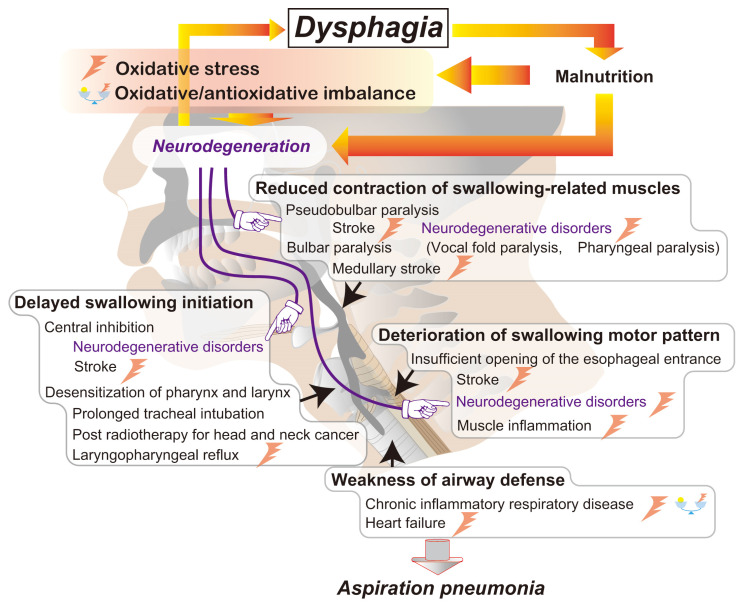
A schematic representation of the pathogenesis of pharyngeal dysphagia linked to oxidative stress, malnutrition, and neurodegeneration. Pharyngeal dysphagia is primarily caused by several related factors, including delayed swallowing initiation, reduced contraction of swallowing-related muscles, deterioration of the swallowing motor pattern, and a weakness of airway defense [62,64,66,86,120,126]. Many diseases causing dysphagia, including neurodegenerative disorders, are also linked to oxidative stress and oxidative/antioxidative imbalance [97,106,113,123,145]. Moreover, malnutrition, possibly resulting from dysphagia, may exacerbate neurodegeneration and oxidative stress levels that are linked to neuron degradation [115,119,120,121,122]. A detrimental feedback loop that worsens nutritional deficits and neurodegenerative processes might influence swallowing function.

Oxidative stress is a key factor in the progression of airway defensive mechanism dysfunction pathogenesis, in which there exists a fundamental link among dysphagia, malnutrition, neurodegeneration, and oxidative stress [119,120]. Dysphagia has been closely linked to inadequate nutritional intake in patients. Moreover, an imbalance of proper nutrition has been found to cause nutritional oxidative stress [121]. Malnutrition has been found to accelerate neurodegeneration [122]. As previously discussed in this paper, oxidative stress is linked to the progression of dysphagia and other airway defense pathologies. In this study, we establish that airway defense pathologies form a sequence of reciprocal causes and effects that aggravate the patient’s pathogenic symptoms through malnutrition-derived oxidative stress and neurodegeneration. Neurons are particularly susceptible to oxidative stress, which is likely linked to the development and exacerbation of neurodegenerative diseases such as Alzheimer’s disease and Parkinson’s disease. Specifically, axons have been proven to produce neuron-specific oxidation [123]. The potential causes of dysphagia and the role of oxidative stress in the decline in swallowing functionality are summarized in Figure 2.

Laryngopharyngeal reflux disease causes irritation in the larynx and airway, which can lead to a persistent cough. Conversely, prolonged exposure of the laryngeal mucosa to gastric acid may result in a sensory deficit in the larynx, accompanied by acid-induced laryngeal edema [124,125,126]. This condition could decrease the swallowing reflex [126]. Oxidative stress may also be linked to chronic acid exposure of the larynx, which is accompanied by reduced levels of antioxidant enzymes such as catalase and glutathione [124,127,128].

Inflammatory or allergic airway sensitization is prevalent in individuals with chronic respiratory disorders, such as asthma, chronic obstructive pulmonary disease (COPD), and chronic bronchitis. Numerous previous reports on oxidative stress in chronic airway diseases in humans and animals have indicated a relationship between chronic airway inflammation and oxidative stress [129,130,131,132,133,134,135,136,137,138,139,140,141,142]. The persistent inflammation in the lower airways elevates oxidative stress levels and diminishes the activity of antioxidant defense enzymes. This imbalance contributes to ongoing inflammatory damage and exacerbates the severity of respiratory conditions [131,135,140]. The intrinsic factor that exacerbates pulmonary inflammation is derived from pulmonary cells, such as epithelial and smooth muscle cells, which can release elevated levels of IL-4, IL-5, IL-10, and IL-13 [139,143]. In addition, the extrinsic influence, including ozone and cigarette smoking, deteriorates these airway diseases, leading to the damage of epithelial cells via secondary mediators, the activation of the signaling pathway, and pollutant-induced reactive oxygen species (ROS) [131,132,138,140,142,144,145]. Cigarette smoking is a significant risk factor for deteriorating airway diseases due to airway irritation. Nicotine is the chemical compound in cigarette smoke that is primarily responsible for airway irritation, which may be linked to the activation of nicotinic acetylcholine receptors (nAChRs) found in the respiratory tract. This activation can trigger airway defense reflexes, including coughing [146]. Moreover, high levels of oxidative stress are a critical factor facilitating neurotoxicity and DNA damage, primarily through their impact on cellular metabolism, mainly as a result of dysfunction in the metabolism of mitochondrial ROS [147,148,149], which leads to the greater sensitivity of the cough reflex by activating transient receptor potential (TRP) channels and protein kinase C [150].

Oxidative damage can affect considerable physiological systems including cardiovascular and metabolic conditions in patients with obstructive sleep apnea, potentially impacting the patency of the respiratory tract [151,152,153]. Repeated episodes of hypoxia and hypercapnia, as well as periods of normal oxygen levels caused by unstable airway patency, are similar to recurrent events of ischemia and reperfusion. These events can lead to elevated levels of various markers associated with oxidative stress and inflammation [151,152,153,154,155]. As such, pulmonary and cardiovascular disorders can significantly compromise swallowing function, increasing the risk of aspiration pneumonia [156,157,158].

## 5. Possible Contributions of Antioxidants to Maintenance of Upper Airway Defensive Reflexes

Research investigating the therapeutic effects of antioxidants on compromised upper airway reflexes and the maintenance of respiratory function has advanced significantly. Nevertheless, the practical application of these findings remains limited.

Edaravone has demonstrated efficacy in addressing cerebral infarction by enhancing nitric oxide (NO) synthesis through its antioxidant properties, which protect the damaged vascular endothelium while inhibiting thrombus formation [106,159]. These pharmacological features provide therapeutic potential in mitigating the functional deficit of ischemic neural injury and promoting recovery. Antioxidant supplements offer promising therapeutic benefits by facilitating the recovery of swallowing functions [98,160]. However, the impact of ROS on the neurodegenerative dysfunction of the swallowing center following reperfusion remains largely unexplored (Figure 2). A recent animal study has shown that Tempol remarkably enhances the activity of the genioglossus muscle in a dose-dependent manner, which suggests that antioxidants could modulate the muscle function of the upper airway [151].

Hydrogen sulfide (H_2_S) could endogenously function as a significant neuromodulator within the brain [97,160]. Likewise, exogenous hydrogen sulfide can offer promising therapeutic benefits, including antioxidant and anti-inflammatory effects: the mitigation of ischemia–reperfusion injuries in the brain, a reduction in hypoxia-induced damage to the respiratory CPG, and the alleviation of airway inflammation linked to oxidative stress [97,103,161,162,163]. A previous animal study has demonstrated that the administration of sodium hydrosulfide (NaHS), a donor of H_2_S, can reduce neuronal chromatolysis in the hypoglossal nucleus and nucleus ambiguus, which are adversely impacted by prenatal exposure to cigarette smoke [163]. Furthermore, NaHS has been shown to reverse neuronal loss and improve motor dysfunction in a Parkinson’s model [97]. The substance improved neurological symptoms subjected to cerebral ischemia and reperfusion following ischemia [103]. These neuroprotective effects are primarily attributable to the antioxidant and anti-inflammatory properties of H_2_S [97,103]. The effects of antioxidants on oxidative neuronal damage in the swallowing center need to be clarified after blood flow is restored from the vertebral or internal carotid arteries following a stroke. Further, additional studies are necessary to investigate the potential benefits of antioxidants on airway patency, potentially leading to valuable therapeutic applications for conditions related to respiratory obstruction [151,164].

Asthma and chronic airway diseases are significant issues with rising morbidity rates. The interactions between genetic and environmental factors significantly influence the development and progression of these chronic inflammatory diseases. Oxidative stress in the upper and lower airways, including the lungs, enhances the inflammatory response linked to asthma and allergies [129,135,136,142,165,166,167]. The significant increase in oxidative stress levels reduces antioxidant defenses and disrupts fundamental cellular regulatory mechanisms, including autophagy, mitophagy, and apoptosis, further deteriorating chronic airway inflammation [142]. The therapeutic benefit of antioxidant supplements may warrant consideration in cases of inadequate dietary intake of antioxidants and high exposure to environmental oxidants (Figure 2).

The potential role of antioxidants in the treatment of allergic and inflammatory respiratory diseases has been explored in previous studies [144,161,168,169,170,171,172]. Several studies have suggested that antioxidant intake during pregnancy may be linked to reduced antioxidant capacity of the respiratory system and a decreased risk of wheezing in infants [145,173]. This association highlights the potential significance of maternal nutrition in promoting an allergic and inflammatory state of the airway.

Allergic rhinitis may be a potential target of antioxidant therapy. Higher malondialdehyde and lower glutathione levels in oral and nasal exhaled breath condensate were observed in patients with allergic rhinitis and asthma [165]. Conversely, vitamin E supplementation did not improve the severity of nasal symptoms or serum-specific IgE levels in patients with allergic rhinitis [170,174]. Antioxidant therapy remains a controversial topic in the treatment of allergic rhinitis and asthma [175,176]. Further studies are necessary using animal models to explore the effects of antioxidants such as Twendee X on the pathogenic immune response and the hypersensitivity of the sneezing reflex, which will probably lead to better therapeutic strategies for managing allergic rhinitis symptoms.

N-acetylcysteine, a synthetic derivative of the endogenous amino acid L-cysteine and a precursor of glutathione, functions as an antioxidant and is a potential candidate for therapeutic treatment of various diseases, including inflammatory and neurodegenerative diseases (e.g., asthma, chronic bronchitis, laryngopharyngeal reflux, and Parkinson’s disease) [177,178,179,180]. Erdosteine, an *N*-(carboxymethylthioacetyl)-homocysteine thiolactone, functions primarily as a mucolytic agent for chronic pulmonary diseases, but it also acts as an effective free radical scavenger [181]. Its antioxidant effect likely prevents the viability of motor neurons from ischemic and reperfusion injury [181,182,183]. In addition, the antioxidant and anti-inflammatory activities of Erdosteine may significantly mitigate allergic inflammation by reducing the production of ROS [184]. Further investigations are needed to assess how these antioxidants contribute to the functional improvement in airway protective reflexes, such as post-stroke dysphagia and the cough and sneeze reflex hypersensitivity primarily caused by allergic respiratory disorders.

## 6. Conclusions and Perspectives

The mechanisms that govern airway defense reflexes and their integration with the respiratory process are complex and not yet fully understood (Figure 1). Neuronal regulation, proper muscle contraction, and the optimal condition of peripheral organs—such as the larynx and lower airways—are linked to oxidative stress in a specific way. Increased oxidative stress and an imbalance between oxidative and antioxidative status can complicate airway disorders, including impaired airway clearance and defensive reflexes, oropharyngeal dysphagia, and chronic cough (Figure 2). Although the clinical significance of antioxidant therapy in optimizing airway defensive reflexes is likely relevant, limited information has been provided concerning therapeutic strategies for dysphagia, chronic cough, and allergic rhinitis. Further studies using animal models are warranted to elucidate the role of oxidative stress in the pathophysiology of these disorders. Moreover, clinical trials are also necessary to assess the effectiveness of antioxidants in reducing symptoms related to impaired airway defensive reflexes.

## Abbreviations

The following abbreviations are used in this manuscript:

AEN	anterior ethmoidal nerve
ALS	amyotrophic lateral sclerosis
BötC	Bötzinger complex
CNS	central nervous system
COPD	chronic obstructive pulmonary disease
CPG	central pattern generator
cVRG	caudal ventral respiratory group
DSG	dorsal swallowing group
E-AUG	augmenting expiratory neuron
E-DEC	decrementing expiratory neuron
E-M	expiratory motoneuron
GPN	glossopharyngeal nerve
H_2_S	hydrogen sulfide
I-AUG	augmenting inspiratory neuron
I-DEC	decrementing inspiratory neuron
I-E	Inspiratory–expiratory neuron
I-M	inspiratory motoneuron
MN	cranial motoneuron
NaHS	sodium hydrosulfide
NO	nitric oxide
NTS	nucleus tractus solitarius
PBN/KF	parabrachial/Kölliker–Fuse nuclei
P-cell	pump neuron
PiCo	post-inspiratory complex
pre-BötC	pre-Bötzinger complex
pre-I/I	pre-inspiratory/inspiratory neuron
RAR	rapidly adapting receptor
RLN	recurrent laryngeal nerve
ROS	reactive oxygen species
RTN/pFRG	retrotrapezoid nucleus/parafacial respiratory group
rVRG	rostral ventral respiratory group
SAR	slowly adapting receptor
SLN	superior laryngeal nerve
TRP	transient receptor potential
TRPV1	transient receptor potential vanilloid 1
VN	vagus nerve
VSG	ventral swallowing group
